# Identification of a novel anti-σ^E ^factor in *Neisseria meningitidis*

**DOI:** 10.1186/1471-2180-10-164

**Published:** 2010-06-04

**Authors:** Carla Th P Hopman, Dave Speijer, Arie van der Ende, Yvonne Pannekoek

**Affiliations:** 1 Academic Medical Center, Center for Infection and Immunity Amsterdam (CINIMA), Department of Medical Microbiology, Amsterdam, the Netherlands; 2 Academic Medical Center, Department of Medical Biochemistry, Amsterdam, the Netherlands

## Abstract

**Background:**

Fine tuning expression of genes is a prerequisite for the strictly human pathogen *Neisseria meningitidis *to survive hostile growth conditions and establish disease. Many bacterial species respond to stress by using alternative σ factors which, in complex with RNA polymerase holoenzyme, recognize specific promoter determinants. σ^E^, encoded by *rpoE *(NMB2144) in meningococci, is known to be essential in mounting responses to environmental challenges in many pathogens. Here we identified genes belonging to the σ^E ^regulon of meningococci.

**Results:**

We show that meningococcal σ^E ^is part of the polycistronic operon NMB2140-NMB2145 and autoregulated. In addition we demonstrate that σ^E ^controls expression of methionine sulfoxide reductase (MsrA/MsrB). Moreover, we provide evidence that the activity of σ^E ^is under control of NMB2145, directly downstream of *rpoE*. The protein encoded by NMB2145 is structurally related to anti-sigma domain (ASD) proteins and characterized by a zinc containing anti-σ factor (ZAS) motif, a hall mark of a specific class of Zn^2+-^binding ASD proteins acting as anti-σ factors. We demonstrate that Cys residues in ZAS, as well as the Cys residue on position 4, are essential for anti-σ^E ^activity of NMB2145, as found for a minority of members of the ZAS family that are predicted to act in the cytoplasm and responding to oxidative stimuli. However, exposure of cells to oxidative stimuli did not result in altered expression of σ^E^.

**Conclusions:**

Together, our results demonstrate that meningococci express a functional transcriptionally autoregulated σ^E ^factor, the activity of which is controlled by a novel meningococcal anti-σ factor belonging to the ZAS family.

## Background

RNA polymerase holoenzyme, consisting of a 5-subunit core RNA polymerase (α_2_ββ'ω) and a dissociable subunit, sigma (σ), initiates bacterial transcription. The σ factor contains many of the promoter recognition determinants and several σ factors each recognizing their specific class of promoter sequences have been described [1-5]. In general, in exponentially growing bacteria transcription is initiated by RNA polymerase carrying the housekeeping σ, known as σ^70 ^[6]. Alternative σ factors mediate transcription of regulons activated under specific environmental conditions [7,8]. The activity of many alternative σs is inhibited by a specific anti-σ factor. In a wide variety of bacterial species the σ factor σ^E,^, also known as extracytoplasmic factor or ECF, belonging to the group IV σs, is essential in mounting responses to environmental challenges such as oxidative stress, heat shock, and misfolding of membrane proteins [9,10]. In addition, σ^E ^is of importance for virulence of bacterial pathogens [11-22]. The regulon size of σ^E ^varies widely among bacterial species studied, ranging from 89 unique σ^E ^controlled transcription units in *E. coli *and related bacteria [23] to a relatively small regulon of 5 genes in *Neisseria gonorrhoeae *[24]. In most examples, the gene encoding σ^E ^(*rpoE*) is located in an autoregulated operon that also contains, directly downstream of *rpoE*, the gene encoding its cognate anti-σ^E ^factor [25-28]. Extensive sequence analysis showed that about one third (1265/˜3600) of known and predicted anti-group IV σ factors, encoded in a gene cluster with a group IV σ (with only one exception), contain a conserved structural N-terminal fold, recently described as the anti-sigma domain (ASD) [26]. Typically, the ASD is in the N-terminus, oriented towards the cytoplasm, preceding a C-terminal transmembrane segment. However, 20% of the 1265 ASD containing proteins are not predicted to contain a transmembrane spanning C-terminal domain [26]. Among these, 95% (227/248) are characterized by the presence of an invariant Hisx_3_Cysx_2_Cys sequence motif important for anti-sigma activity, co-ordinating Zn^2+^, described as the zinc containing anti-σ factor (ZAS) group IV anti-σs proteins [29]. ASD proteins and ASD proteins containing the ZAS motif are predicted to bind specifically to σs and inhibit their activities [25-28].

The strictly human pathogen *Neisseria meningitidis *colonizes the nasopharynx of approximately 10 to 30% of the population. In rare instances colonization results in invasive disease leading to life-threatening septicemia and meningitis [30]. Meningococci possess a variety of genes involved in adaptation to specific changes in the environment encountered in the host [31-36]. In addition to nutrient limitation, meningococci are also exposed to massive amounts of reactive oxygen species produced by host defenses [37,38]. Fine tuning expression of genes required to survive hostile growth conditions is a prerequisite for the meningococcus to establish disease.

All four publicly available, completely sequenced genomes of *N. meningitidis *contain a gene (NMA0233, NMB2144, NMC2123 and NMCC˜2103) encoding a protein with homology to σ^E^, the σ factor involved in stress responses [39-42]. In this study we explored the σ^E ^regulon of *N. meningitidis*. In addition, we provide evidence that the expression of σ^E ^(encoded by NMB2144) in meningococci is autoregulated and that its activity is under control of a protein encoded directly downstream of *rpoE*. This protein, encoded by NMB2145, is structurally related to ASD proteins and contains the ZAS motif (His30x_3_Cys34x_2_Cys37). We demonstrate that the Cys residues in the ZAS motif, as well as a Cys on position 4, are important (Cys4 and C37) or essential (Cys34) for anti-σ^E ^activity of NMB2145.

## Results

### The gene cluster containing *rpoE *is transcribed as a polycistronic operon and transcriptionally regulated by σ^E^

**Figure 1:**
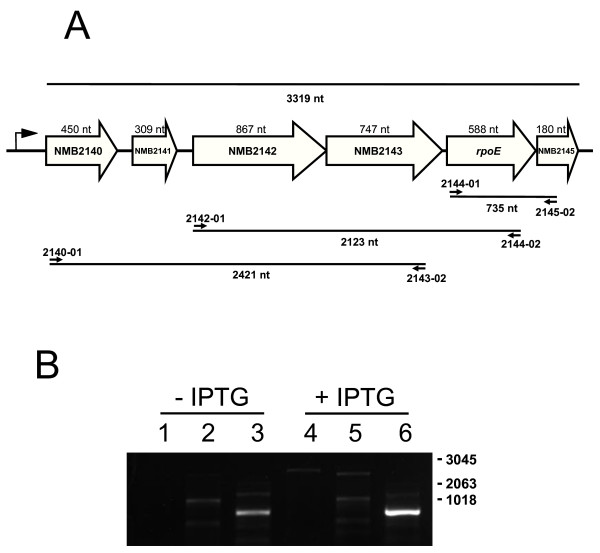
**Transcriptional analysis of the NMB2140-NMB2145 region**. **A) **Schematic representation of the organization of the NMB2140-NMB2145 region. Genes are indicated as open arrows that show the orientation and relative sizes of the putative ORFs. Primers used in RT-PCR are indicated by closed arrows. Sizes of calculated RT-PCR products are indicated below the black lines. The bent arrow indicates the promoter. **B) ***RpoE *is cotranscribed in the polycistronic operon NMB2140-2145 upon overexpressing of *rpoE*. RT-PCR analysis of transcription of the *rpoE *operon in H44/76 transformed with pNMB2144 before (- IPTG) and after (˜IPTG) induction of over expression of *rpoE*. Products obtained by RT-PCR were separated on agarose gels. Numbers on the right represent DNA marker sizes; lanes 1, 4,: RT-PCR product (calculated size 2421 nt) obtained with primer pair 2140-01/2143-02; lanes 2, 5,: RT-PCR product (calculated size 2123 nt) obtained with primer pair 2142-01/2144-02; lanes 3, 6,: RT-PCR product (calculated size 735 nt) obtained with primer pair 2144-01/2145-02. Reactions without reverse transcriptase did not yield any products (not shown).

In many bacterial species, *rpoE *is part of an autoregulated polycistronic operon also encoding its cognate anti-sigma factor [25-28]. In meningococci, NMB2144 is annotated as *rpoE*, encoding a protein with a molecular weight of approximately 23 kDa, 98% identical to the σ^E ^orthologue of *N. gonorrhoeae *[24] and 28% identical to σ^E ^of *E. coli*. Meningococcal *rpoE *is part of a ˜3 kb cluster of genes NMB2140 through NMB2145 (Fig.1a) having a genomic arrangement similar to that found in *N. gonorrhoeae *[24]. All genes, except NMB2144, are annotated as hypothetical proteins. The minimal spacing found in the cluster suggests co-transcription of its genes.

To investigate whether the genes found in the gene cluster NMB2140 through NMB2145 are co-transcribed and under transcriptional control of σ^E^, a meningococcal strain in which expression of *rpoE *can be controlled, was generated by transformation of H44/76 with the shuttle vector pEN11 carrying *rpoE *under control of an IPTG-inducible promoter, creating H44/76 + pNMB2144. Transcript levels of the gene cluster were analysed by RT-PCR using RNA isolated from these cells grown in the absence and presence of IPTG and primer pairs as depicted in Fig. 1a. With either wt cells (not shown) or H44/76 + pNMB2144 cells grown in the absence of IPTG, hardly any detectable RT-PCR products of co-transcripts were found (see Fig. 1B, lane 1 to 3). Only the small 735 nt product (NMB2144-NMB2145, see Fig. 1B, lane 3) could be seen (the band in lane 2 is an unrelated product as shown by sequence analysis). In contrast, only when H44/76 + pNMB2144 cells were grown in the presence of IPTG, specific RT-PCR products, with sizes corresponding to calculated sizes (2412 nt (Fig. 1B, lane 4) and 2123 nt (Fig. 1B, lane 5) containing the predicted sequences of NMB2140-NMB2144, were detected, while the 735 nt product was strongly induced (Fig. 1B, lane 6). These observations indicate that the gene cluster containing *rpoE *is transcribed as a polycistronic operon and transcriptionally regulated by σ^E^. The fact that complete transcripts of the *rpoE *operon were only found upon overexpression of *rpoE *suggests that in H44/76 wt cells, under the growth conditions tested, the levels of (active) σ^E ^allow only barely detectable transcription.

### Identification of proteins under control of σ^E^

**Figure 2:**
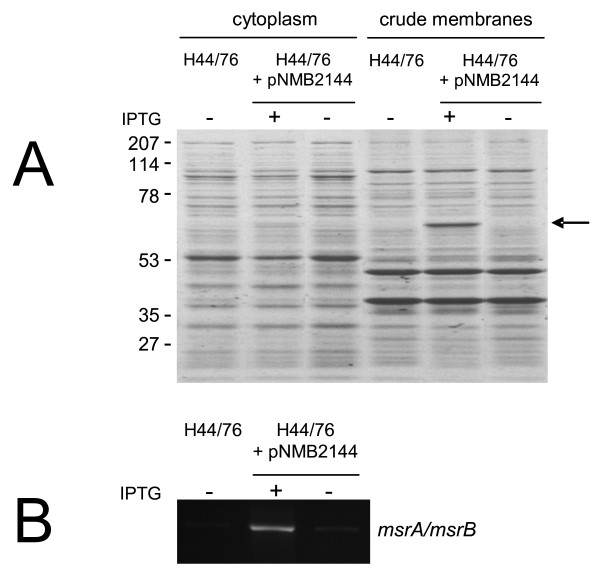
**MsrA/MsrB is induced upon overexpression of *rpoE *via transcriptional control**. Protein analysis of the cytoplasmic and crude membrane fraction by SDS-PAGE (A) and corresponding transcriptional analysis of *msrA/mrsB *by RT-PCR (B) of the wt strain (H44/76) and H44/76 transformed with pNMB2144 before (-) and after induction (+). Molecular weight markers (in kDa) indicated on the left. Arrow indicates MsrA/MsrB.

To further explore the meningococcal σ^E ^regulon, protein patterns of the H44/76 wt strain, Δ*rpoE *and H44/76˜pNMB2144 were compared by SDS-PAGE. No apparent protein expression level differences between H44/76 wt and Δ*rpoE *were observed in the proteomes of the cells (not shown). The addition of IPTG to the culture medium of cells transformed with pNMB2144 only gave minor changes in protein expression in the cytoplasm (Fig. 2a). In contrast, in the crude membrane fraction, a dramatic increase in the expression of a ˜60 kDa protein was observed (Fig. 2a). The increase in expression of this protein was IPTG dependent as the protein was hardly detectable in crude membranes prepared from the same cells not exposed to IPTG (Fig. 2a). Peptide mass fingerprinting, using MALDI-TOF MS to analyze the tryptic fragments generated by in gel digestion, identified this protein as the methionine sulfoxide reductase MsrA/MsrB, encoded by NMB0044 (Mowse score: 191 with 14 matching peptides).

### MsrA/MsrB is transcriptionally controlled by σ^E^

To ascertain that *msrA/msrB *is under direct control of σ^E^, transcript levels of *msrA/msrB *in diverse meningococcal genetic backgrounds were analyzed by RT-PCR using RNA isolated from cells grown in the absence and presence of IPTG and primers targeting *msrA/msrB*. When H44/76 wt or H44/76 + pNMB2144 cells were grown in the absence of IPTG, no detectable RT-PCR products were observed. In contrast, when H44/76 + pNMB2144 cells were grown in the presence of IPTG, an RT-PCR product with a size indicative of transcription of *msrA/msrB *was found (Fig. 2b). The identity of the transcript was confirmed by sequencing of the RT-PCR product. These results strongly suggest that *msrA/msrB *is transcriptionally controlled by σ^E^.

### NMB2145 inhibits transcription of the *rpoE *regulon

One possible explanation for low σ^E ^activity in H44/76 wt cells under the growth conditions tested is that σ^E ^is kept in an inactive state through an interaction with an anti-σ factor, thereby preventing σ^E ^binding to core RNA polymerase, one of the ways to inhibit σ activity found in σ-regulator circuits in other bacteria [43-47]. Interestingly, it was recently reported that NMB2145 contains the ZAS motif Hisx_3_Cysx_2_Cys [48], characteristic for a subset of group IV σ anti-σ factors, usually encoded directly downstream of *rpoE *and cotranscribed [26].

**Figure 3:**
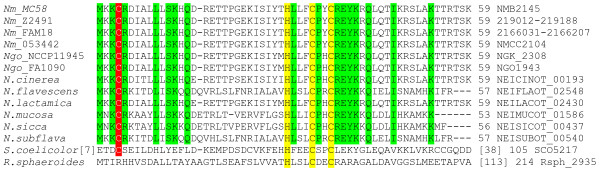
**Sequence alignment of orthologues of NMB2145 as found in other neisserial species and the ZAS containing anti-σ factors RsrA of *S. coelicolor *and ChrR of *R. sphaeriodes***. Numbers at the end of each sequence represent the total length of the protein and bracketed numbers show the number of residues not shown in the alignment of RsrA and ChrR with NMB2145. The ZAS motif (Hisx_3_Cysx_2_Cys) is indicated in yellow, the additional zinc ligand [48] is indicated in red. Conserved residues of neisserial NMB2145 orthologues are indicated in green. Protein IDs or genomic coordinates (in case of missing protein annotation) are indicated on the right. Details regarding strains of which sequences were obtained are listed in the Materials and Methods section.

Amino acid sequence comparison of orthologues of NMB2145 in genomes of three other meningococcal strains, two gonococcal strains and six commensal neisserial species (*N. cinerea, N. flavescence, N. lactamica, N. mucosa, N. sicca and N. subflava*) revealed that the region containing the ZAS motif, as well as the region around Cys4, are highly conserved in these neisserial orthologues of NMB2145. This in contrast with other much less well conserved parts, highlighting the importance of the conserved regions (Fig. 3). The relative positions of the Cys residue and the ZAS motif in NMB2145 (Cys4; His30, Cys34 and Cys37) correspond exactly with those of the Cys residue and the ZAS motif in RsrA (Cys11; His37, Cys41 and Cys44), the anti-σ^R ^factor of *Streptomyces coelicolor*, of which the Cys residues, but not His37, are essential for anti-σ activity of the protein [29] (Fig. 3). These observations suggest that NMB2145 codes for the meningococcal anti-σ^E ^factor.

To test this hypothesis we first investigated the effect of deletion or overexpression of NMB2145 on transcript levels of the *rpoE *operon. To this end, a NMB2145 deletion mutant (ΔNMB2145) was constructed and complemented with NMB2145 using pEN11 carrying NMB2145 under control of an IPTG-inducible promoter (generating ΔNMB2145 + pNMB2145). Transcript levels of the *rpoE *operon were assessed by semi-quantitative RT-PCR using primers annealing to NMB2140 and NMB2143, respectively.

**Figure 4:**
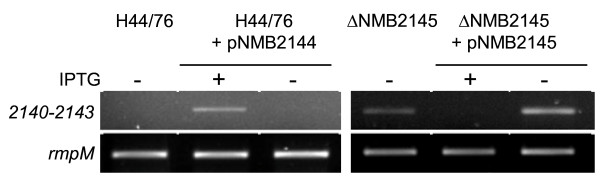
**NMB2145 represses transcription of the *rpoE *operon**. Products obtained by RT-PCR were separated on agarose gel. RT-PCR analysis of transcription of the *rpoE *operon in the wt strain (H44/76), H44/76 transformed with pNMB2144 before (-) and after (+) induction of expression of *rpoE*, after deletion of NMB2145 (ΔNMB2145) and before (-) and after (+) induction of NMB2145 in the ΔNMB2145 background (upper panel). RT-PCR was carried out using primer pair 2140-01/2143-02 (cf Fig.1a) and RT-PCR on *rmpM *(lower panel) was used as input control of total RNA.

As noticed before, RT-PCR products derived from the transcript encoding MsrA/MsrB (Fig.2b) and products indicative of co-transcription of NMB2140-NMB2145 (Fig.1b and Fig. 4) were found only upon overexpression of *rpoE *in trans. However, deletion of NMB2145 resulted in the direct detection of the NMB2140-2143 without the need for overexpression of *rpoE *in the H44/76 wt background. As expected, upon complementation of the ΔNMB2145 mutant by induction of expression of NM2145 in trans, the NMB2140-2143 RT-PCR product was no longer detectable. This effect was dependent upon induction of overexpression of NMB2145 in ΔNMB2145 as it was not observed in the absence of IPTG (Fig.4).

**Figure 5:**
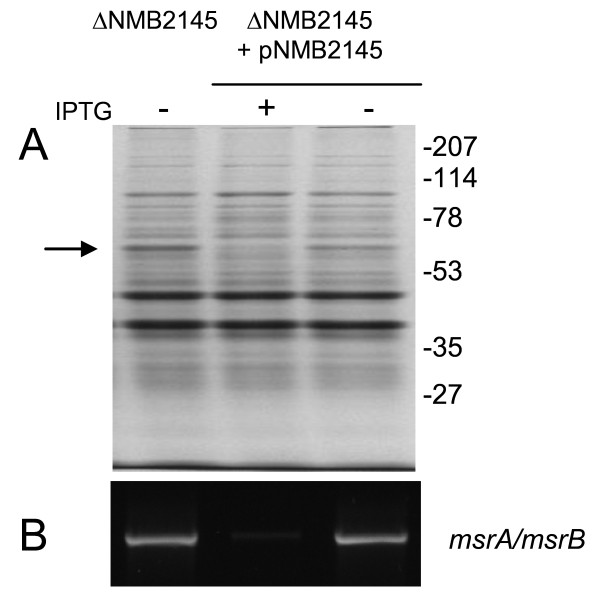
***MsrA/msrB *is expressed upon deletion of and transcriptionally repressed by NMB2145**. Protein analysis of crude membranes by SDS-PAGE (A) and corresponding transcriptional analysis of *msrA/msrB *by RT-PCR (B) of the NMB2145 knockout (ΔNMB2145) and ΔNMB2145 transformed with pNMB2145 before (-) and after induction (+). Molecular weight markers (kDa) are indicated on the right. Arrow indicates MsrA/MsrB.

As one would predict, MsrA/MsrB protein was detected in ΔNMB2145 and could not be detected anymore upon complementation of ΔNMB2145 by NMB2145 when IPTG was added to the culture medium (Fig. 5a). Also, NMB0044 (*msrA/msrB*) RT-PCR product was indeed detected in ΔNMB2145 cells but hardly in ΔNMB2145 cells when complemented by NMB2145 (Fig. 5b).

Together, these experiments demonstrate that NMB2145 inhibits transcription of the *rpoE *regulon. Conceivably, NMB2145 binds to σ^E^, thereby inactivating it, resulting in decreased transcription by means of autoregulation of the *rpoE *operon and, as a consequence of that, decreased transcription of *msrA/msrB*.

### The residues Cys4, Cys34 and Cys37 of NMB2145 are essential for optimal anti-σ^E ^activity

**Figure 6:**
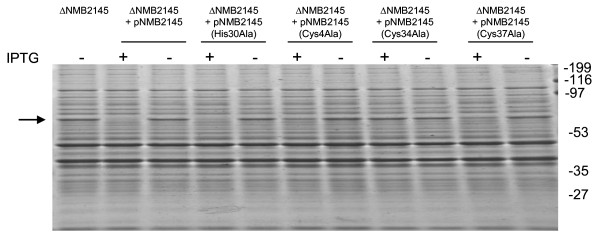
**Residues Cys4, Cys34 and Cys37 of NMB2145 are essential for optimal anti-σ^E ^activity of NMB2145**. SDS-PAGE assessment of MsrA/MsrB protein levels in crude membranes extracted from ΔNMB2145 cells in which mutant NMB2145 proteins pNMB2145(His30Ala), pNMB2145(Cys4Ala), pNMB2145(Cys34Ala) and pNMB2145(Cys37Ala) are expressed. Crude membranes were extracted before (-) and after (+) induction. Molecular weight markers (kDa) are indicated on the right. Arrow indicates MsrA/MsrB.

To investigate whether the Cys residues of the ZAS motif and the conserved Cys at position 4 of NMB2145, in analogy to corresponding Cys residues in RsrA of *S. coelicolor *[29], are also essential for anti-σ^E ^activity of NMB2145, we generated single Ala substitutions at each of the Cys residues and also of the single His residue of the ZAS motif (His30x_3_Cys34x_2_Cys37) and at position 4 of NMB2145. The ability of these mutant NMB2145 proteins to inhibit σ^E ^activity in meningococci was investigated by SDS-PAGE assessment of crude membranes, using MrsA/MrsB as reporter protein. All substitutions except His30Ala resulted in expression of MrsA/MrsB (MALDI-TOF confirmed). The substitution Cys34Ala resulted in MsrA/MsrB levels comparable to those found in crude membranes prepared from ΔNMB2145 cells while the substitutions Cys4Ala and Cys37Ala resulted in more modest, but clearly detectable levels of MsrA/MsrB (Fig. 6). Collectively, these experiments demonstrate that the Cys residues of the ZAS motif, as well as Cys4 of NMB2145 are important for functionality of NMB2145 as an anti-σ^E ^factor.

### Involvement of σ^E ^in the response to hydrogen peroxide, diamide and singlet oxygen

The Cys4 and Cys37 in NMB2145, essential in anti-σ^E ^activity, correspond exactly with Cys11 and Cys44 residues of RsrA of *S. coelicolor *involved in disulphide bond formation. In addition, residue His30 in the ZAS motif of NMB2145 is not required for anti-σ^E ^activity consistent with anti-σ properties of RsrA [29] and ChrR, the ZAS containing anti-σ^E ^factor of *Rhodobacter sphaeroides *[26,49,50]. In *S. coelicolor*, exposure to superoxide, hydrogen peroxide or the thiol specific oxidant diamide causes dissociation of the σ^R^-RsrA complex [46,51,52]. In contrast, ChrR anti-σ^E ^activity is not affected by these reactive oxygen species, but responds to singlet oxygen (^1^O_2_) [53].

To investigate stimuli activating the σ^E ^response in meningococci, cells were exposed to hydrogen peroxide, diamide or singlet oxygen and transcript levels of *rpoE *and *msrA/msrB *were analysed by RT-PCR before and after exposure to these stress agents. No differences in transcript levels of either *rpoE *or *msrA/msrB *were detected, suggesting that in meningococci σ^E ^is not involved in the response to such stimuli. In addition, no detectable differences in transcription levels of *rpoE *and *msrA/msrB *were observed after exposure of cells to SDS-EDTA, a stimulant known to induce membrane stress and activate RpoE in other bacterial species (not shown).

### *In silico *genome wide search for additional genes under control of σ^E ^using a deduced neisserial σ^E ^promoter consensus sequence

Each σ factor recognizes specific promoter sequences, characterized by relatively highly conserved -35 and -10 upstream DNA sequences. Using the promoter sequences of genes under the control of σ^E^, a consensus sequence can be deduced. In several bacterial species, this motif has been successfully used for *in silico *genome searches to identify genes putatively controlled by σ^E^. The σ^E ^dependent transcription of these genes can subsequently be confirmed by *in vitro *experiments [23,54-56].

**Figure 7:**
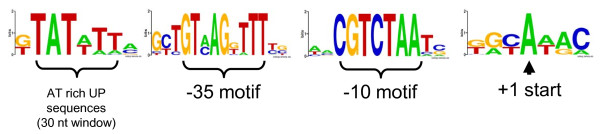
**Consensus promoter sequences predicted to be recognized by σ^E^**. Consensus sequence logo's of the A/T rich UP sequence, the -35 and -10 motif and the +1 start obtained from the compilation of DNA sequences of orthologues of NMB044 and NMB2140 of 12 different neisserial strains. Letter heights indicate the frequency with which a given base is represented at each position. The spacing between the -35 and -10 motifs is 12-13 nt (not shown). Sequence logo's were generated using Weblogo http://weblogo.berkeley.edu/:[57]

To further explore the meningococcal σ^E ^regulon, we used a similar strategy. However, in the meningococcus we were able to demonstrate transcriptional control by σ^E ^for only one operon (the *rpoE *operon itself) and one gene (*msrA*/*msrB*), so far. Therefore, we extended the number of genes from which a σ^E ^promoter consensus sequence could be deduced with orthologues of NMB2140 and NMB0044 found in the sequences of 3 other meningococcal genomes, 2 gonococcal genomes and the genomes of 6 commensal neisserial species. In total, putative promoter sequences of 24 genes were used to generate a consensus promoter sequence by Weblogo [57]. Thus, the conserved putative -35 (GTMAGBWTT) and -10 (CGTCTAAH) motifs could be identified (Fig.7). These motifs are separated by spacer of 12-13 nt (not shown). In addition, an AT rich sequence was observed ˜30 nt upstream of the -35 motif, corresponding to a consensus sequence designated the UP element [58-60]. Six nucleotides downstream of the -10 motif a highly conserved adenosine is found. This nucleotide and its position correspond exactly with the transcriptional start as experimentally identified for *msrA*/*msrB *in gonococci [24].

Next, we used the -35 and -10 motif, allowing a spacer length of 10 to 16 nt to explore the genome of *N. meningitidis *MC58 to identify genes containing the *rpoE *promoter motif. Besides NMB2140 and NMB0044, no other genes were identified.

## Discussion

According to the annotation of the genome of *N. meningitidis *four genes are supposed to encode σ factors: *rpoD *(σ^70^), *rpoH *(σ^32^), *rpoN *(σ^54^) and *rpoE *(σ^E^) [24,39-42]. To our knowledge, so far no information is available regarding the functionality of alternative σ factors in the meningococcus. Here, we describe the first detailed investigation of the functionality of σ^E ^of *N. meningitidis*. In addition, we provide strong evidence that NMB2145, encodes a novel anti-σ factor structurally related to ASD proteins and containing the ZAS motif, making NMB2145 the first anti-σ-factor described for any neisserial species.

Experimental evidence for transcriptional control by σ^E ^could be provided for only 7 genes, the 6 gene containing σ^E ^operon and *msrA/msrB*. In line with this, genome wide *in silico *searches for genes with a σ^E ^promoter motif also did not result in additional genes putatively controlled by σ^E^. This suggests a surprisingly small σ^E ^regulon in meningococci, as well as in gonococci [24] as compared to that of other bacterial species as σ^E ^regulons can comprise up to 89 transcription units (in *E. coli *K-12 and related bacteria) [23].

Although the consensus σ^E ^promoter recognition motifs of the -35 region of meningococci (GTAAGGTT) and *E. coli *(GGAACTT) are quite different, the last 5 residues of the -10 motifs of meningococci (TCTAA) and *E. coli *(TCAAA) differ in only one nucleotide [23]. In addition, other similarities between the structural elements of these promoter regions were observed, such as the AT rich sequence ˜30 nt upstream of the -35 motif. This sequence, designated the UP-element, is a binding site for the C-terminal domain of the α-subunit of RNA polymerase [58-60] and has recently been shown to increase transcription of σ^E ^dependent promoters [61].

Recently, the first comprehensive analysis of conservation and variation of the σ^E ^regulon in *E. coli *and related organisms was reported [23]. The products of the core genes of the conserved σ^E ^regulon coordinate assembly and maintaince of lipopolysaccharide (LPS) and outer membrane proteins (OMPs) of Gram-negative bacteria, in response to cell envelope stress. The majority of the variable regulon members are functionally involved in pathogenesis [23]. Of interest, it was also recently demonstrated that σ^E ^promoters in *E. coli *and its close relatives exhibit a large dynamic range, with a few strong and many weak promoters [61]. The three strongest promoters all carry out regulatory roles in the σ^E ^response, the strongest transcribing σ^E ^itself and its negative regulators, and the next two strongest transcribing small RNAs (sRNAs) involved in downregulation of porin expression [61]. We did not observe expression differences of major outer membrane proteins upon overexpression of σ^E^, making the involvement of σ^E ^in meningococci cell envelope stress uncertain. This was confirmed by our observation that membrane stress did not alter σ^E ^activity. However, as mentioned before, the majority of σ^E ^dependent proteins are expressed at low levels [61], which might be below the detection limit of the assay used in this study. As it is much easier to detect small changes in the transcriptome comparing Δ*rpoE *(σ^E ^knock out) or H44/76 + pNMB2144 (σ^E ^overexpression) with H44/76 (wt strain), we are planning those experiments. The recent identification in *N. meningitidis *of an sRNA controlling a gene and functional Hfq facilitating the interaction between sRNA and target mRNA, suggests the existence of a ribo-regulated network in this pathogen [62-65]. In many other species links between the σ^E ^regulon and the ribo-regulated network exist [66-71], but in meningococci this is as yet unexplored.

The genetic organization of the *rpoE *operon (NGO1948 through NGO1943) of *N*. *gonorrhoeae *is identical to that of meningococci (NMB2140-NMB2145), and four genes, NGO1946, NGO1947, NGO1948 belonging to the *rpoE *operon, and NGO2059, encoding MsrA/MrsB, were also upregulated, along with σ^E ^(NGO1944) itself, in a gonococcal strain overexpressing *rpoE *[24]. We demonstrated cotranscription of all genes in the meningococcal *rpoE *operon. The function of proteins encoded by NMB2140-NMB2143 is currently unknown. NMB2140 might encode a protein with possible trans membrane domains and contains motifs found in the DoxX/D-like family, involved in oxidation of sulfur [72,73]. NMB2141 through NMB2143 encode hypothetical proteins of unknown function.

Based on the structural relatedness of NMB2145 to ASD proteins [26] and sequence conservation of Cys residues shown to be essential for anti-σ^R ^activity of RsrA of *S. coelicolor *[29] we argue that NMB2145, directly downstream of and co-transcribed with *rpoE*, encodes the anti-σ^E ^factor. Indeed, upon deletion of NMB2145, *msrA*/*msrB*, which we demonstrated to be transcriptionally controlled by σ^E^, was abundantly expressed. Irrefutable evidence for a functional interaction of NMB2145 with σ^E ^was obtained by the substitution of Cys residues with Ala at positions in NMB2145 that correspond to Cys residues in RsrA. We found that Cys34 of NMB2145 is essential and, albeit to a lesser extent, Cys4 and Cys37 are also required for optimal anti-σ^E ^activity of NMB2145. We therefore suggest annotating NMB2145 as MseR, Meningococcal sigmaE Regulator.

RsrA is a metalloprotein, containing near-stoichiometric amounts of Zn^2+ ^[29]. Oxidation induces a disulphide bond between two of the Zn^2+ ^ligands (Cys11 and Cys44) resulting in loss of Zn^2+ ^and dissociation of the σ^R^-RsrA-complex, thereby allowing σ^R ^transcription. Thioredoxin is able to reduce oxidized RsrA, and the induction of expression of thioredoxin itself is σ^R ^dependent, suggesting that σ^R^, RsrA and the thioredoxin system in *S. coelicolor *comprise a novel feedback homeostasis loop, sensing and responding to changes in the intracellular thiol-disulphide redox balance [29,52,74,75]. The Cys4 and Cys37 in NMB2145, of importance in anti-σ^E ^activity, correspond exactly with Cys11 and Cys44 residues of RsrA involved in disulphide bond formation, suggesting that MseR also contains Zn^2+^. Therefore, it was tempting to speculate that a similar thiol-disulphide redox balance also exists in meningococci. However, in *N. meningitidis *thioredoxin appears not to be upregulated upon exposure to hydrogen peroxide [34] and we showed that transcription levels of MsrA/MsrB are not affected after exposure of meningococci to hydrogen peroxide, diamide or singlet oxygen. Whether NMB2145 is also a Zn^+ ^containing protein, deserves further study.Together, despite the structural resemblance between RsrA and MseR, these results show that MseR functionally differs from RsrA of *S. coelicolor*.

MsrA/MrsB, encoding methionine sulfoxide reductase, an enzyme repairing proteins exposed to reactive oxygen species [76], is a major target of σ^E^, and abundantly expressed when active σ^E ^levels are high. Expression of MsrA/MsrB is also controlled by σ^E ^in *N. gonorrhoeae *and *Caulobacter crescentus*. Interestingly, in *N. gonorrhoeae *MsrA/MsrB is upregulated together with the genes NGO1947 and NGO1948 in response to hydrogen peroxide [24,77,78]. However, none of the meningococcal orthologues [34,78], nor σ^E ^activity, as shown in our study, appear to respond to hydrogen peroxide,strongly indicating the existence of different modes of regulation of σ^E ^between gonococci and meningococci. In addition we did not found detectable differences in transcription levels of MsrA/MsrB after exposure to SDS-EDTA, a stimulant known to activate RpoE in other bacterial species. Thus, *in vivo *stimuli activating the σ^E ^response in *N. meningitidis *are most likely different from those of gonococci and remain to be further explored.

## Conclusions

The results show the existence of a σ^E ^regulon in meningococci. The product of NMB2145 (MseR) functions as an anti-σ^E ^factor with properties different from membrane spanning anti-σ^E ^factors responding to signals in the periplasma. Our data strongly indicate that MseR, the meningococcal anti-σ^E ^factor, closely mimics structural properties of members of the ZAS family that are acting on novel stimuli encountered in the cytoplasm. Stimuli of MseR differ from those of the ZAS family anti-sigma factors suggesting that MseR is a novel anti-σ factor. This could indicate a potentially important, specific role for σ^E ^in the pathogenesis of meningococcal disease.

## Methods

### Bacterial strains and culture conditions

*N. meningitidis *strain H44/76, B: P1.7,16: F3-3: ST-32 (cc32), is closely related to the sequenced serogroup B strain MC58, belonging to the same clonal complex [79]. Meningococci were grown on GC plates (Difco) supplemented with 1% (vol/vol) Vitox (Oxoid) at 37°C in a humidified atmosphere of 5% CO_2_. Broth cultures were incubated in tryptic soy broth (BD) on a gyratory shaker (180 rpm) at 37°C. When appropriate, plates or broths were supplemented with erythromycin (Erm) (5 μg/ml) and/or chloramphenicol (Cm) (5 μg/ml). Growth was monitored by measuring optical density of cultures at 600 nm (OD_600_) at regular time intervals. To investigate the effect of various stress agents on RpoE activity, cells were grown to mid log phase (OD_600 _= 0.6-0.7) and treated for different time periods (30 min-1 h) with hydrogen peroxide (5 mM), diamide (100 mM), 0.01% SDS-0.1 mM EDTA or methylene blue (1 μM) in the presence of white light (source of singlet oxygen) [77]. Sequences from the following strains (with Genbank ID) were downloaded for comparative aligments: *N.meningitidis_MC58 *(AE002098); *N.meningitidis*_FAM18 (AM421808); *N.meningitidis*_053442 (CP000381); *N.meningitidis*_Z2491 (AL157959); *N.gonorrhoeae*_FA1090 (AE004969); *N.gonorrhoeae*_NCCP11945 (CP001050); *N.cinerea*_ATCC_14685 (ACDY00000000); *N.flavescens_*NRL30031/H210 (ACEN00000000); *N.lactamica_*ATCC_23970 (ACEQ00000000); *N.subflava*_NJ9703 (ACEO00000000); *N.sicca*_ATCC_29256 (ACKO00000000); *N.mucosa_*ATCC_25996 (ACDX00000000); *Streptomyces coelicolor*_A3(2) (AL645882); *Rhodobacter sphaeroides*_ATCC_17025 (CP000661).

### Construction of Δ*rpoE *and ΔNMB2145 mutants of *N. meningitidis*

*N. meningitidis *H44/76 knock-out mutants of *rpoE *(NMB2144) and NMB2145 were constructed using the PCR-ligation-PCR method [79,80]. All primers used in this study are listed in Table 1. PCR products were generated with primer pairs CTsE-1/CTsE-2 and CTsE-3/CTsE4 for creating Δ*rpoE *and primer pairs CT-2145-1/CT2145-2 and CT-2145-3/CT-2145-4 for creating ΔNMB2145, ligated and the ligation products were reamplified with primer pairs CTsE-1/CTsE-4 (for Δ*rpoE*) and CT-2145-1/CT-2145-4 (for ΔNMB2145). The resulting PCR products were cloned into pCR2.1 (Invitrogen). The EcoRI digested Erm resistance cassette from pAErmC' [81] was introduced into the created unique MfeI restriction site yielding plasmids pCR2.1-NMB2144 and pCR2.1-NM2145. The Δ*rpoE *and ΔNMB2145 strains were generated by natural transformation of strain H44/76 with pCR2.1-NMB2144 and pCR2.1-NMB2145 respectively, and selection for Erm resistance. Replacement of NMB2144 and NMB2145 by the Erm cassette was confirmed by PCR with primer pair CTsE-5/CTsE-6 (for Δ*rpoE*) and primer pair 2144-01/CT-2145-6 for ΔNMB2145. The orientation of the Erm cassette was determined by PCR using primer pair JP19/JP20 and mutant strains in which the transcriptional direction of the Erm cassette was in accordance with the transcriptional direction of the deleted genes were selected.

**Table 1:** Oligonucleotides used in this study

Name	Sequence 5'-3'
CTsE-1	AACAGCCTTGTTAATATGGCG
CTsE-2	ATCACAATTGATATGGCGGACAAGGAG
CTsE-3	CAGGACCGGGAAACCACC
CTsE-4	GAAGTGTGTATTATGGATG
CTsE-5	GCGAGTTCCTCCAATATTGCC
CTsE-6	CGGGAGGGGTAGCGCCG
CT-2145-1	CGCAGGAAGGGCAGCCGC
CT-2145-2	ATCACAATTGCGTTTACTTCGGGTTTTCTTGG
CT-2145-3	ACGGCATAAGCTGACGG
CT-2145-4	CGTTTGAGATTGTGCCG
CT-2145-6	TGGCAGTCTTGTATGCGGG
CTsE-7	ATCATATGCCGCTACCCGACC
CTsE-8	TTCATGAACGTTTACTTCGGG
CT-2145-11F	CGTCATATGAAAAAATGCCGCG
CT-2145-11R	TTTCATGACGGCATTTATTTTGAAGTTCTGG
2140-01	TCGGAACAACTTGGCAG
2143-02	GACCATTTCCCAAGCAA
2142-01	ATGATTCAACACGCAGG
2144-01	ACCCGACCTGACCGATGC
2144-02	GGTGCATAATGGTGTGGT
2145-02	CTGGTTGTTTTTGCCAG
CT-class4-1	CAAACAGCTGAAATTAAGCGC
CT-class4-2	GTGATGATTGTGTGCCGGC
CT-MSR-01	CTTTGCGCCAAGTTCGGC
CT-MSR-02	CTTTACCTTTCAACGCGCC
pEN11F	TGTGGAATTGTGAGCGGATA
pEN11R	AGCAAAAACAGGAAGGCAAA
ALpEN11F2	GACAATTAATCATCGGCTCGT
JP19	TAAATACAAAACGCTCATTGGC
JP22	AAATCGTCAATTCCTGCATGTT
YPNMB2145-C4A-FW	GCATATGAAAAAAGCCCGCGATATCGCCC
YPNMB2145-C4A-RP	GGGCGATATCGCGGGCTTTTTTCATATGC
YPNMB2145-H30A-FW	GATTTCCATATACACAGCCCTGCTGTTCTGTCCG
YPNMB2145-H30A-RP	CGGACAGAACAGCAGGGCTGTGTATATGGAAATC
YPNMB2145-C34A-FW	CACACACCTGCTGTTCGCTCCGTATTGCCGTG
YPNMB2145-C34A-RP	CACGGCAATACGGAGCGAACAGCAGGTGTGTG
YPNMB2145-C37A-FW	GCTGTTCTGTCCGTATGCCCGTGAATATAAAAGAC
YPNMB2145-C37A-RP	GTCTTTTATATTCACGGGCATACGGACAGAACAGC

### Overexpression of *rpoE *and complementation of ΔNMB2145

To overexpress *rpoE *and to complement ΔNMB2145, NMB2144 and NMB2145 of strain H44/76 were amplified with primer pair CTsE-7/CTsE-8 (for NMB2144) and primer pair CT-2145-11F/CT-2145-11R (for NMB2145). Forward primers (CTsE-7 and CT-2145-11F) contain an NdeI restriction site and the reversed primers an RcaI restriction site. The resulting PCR products and shuttle vector pEN11-pldA [82] were digested with NdeI and RcaI, ligated into NdeI-RcaI predigested pEN11-pldA and transformed to *E. coli *TOP10F'(Invitrogen). Cm-resistant colonies were checked by colony PCR and sequencing, using pEN11F, pEN11R and ALpEN11F2 primers. Plasmids of clones containing an intact NMB2144 coding region (pNMB2144) or an intact NMB2145 coding region (pNMB2145) were isolated to transform H44/76 and ΔNMB2145 thereby generating H44/76 + pNMB2144 and ΔNMB2145 + pNMB2145, respectively. Expression of recombinant DNA was induced by addition of IPTG to the culture medium to a final concentration of 1 mM.

### In vitro mutagenesis of NMB2145

Shuttle vector pNMB2145 was used to generate mutant NMB2145 proteins. Point mutations were generated using Quickchange site-directed mutagenesis (Stratagene). Four mutants of pNMB2145 were generated: Cys4Ala, His30Ala, Cys34Ala and Cys37Ala, using primer pairs YPNMB2145C4AFW-YPNMB2145C4ARP (for mutant pNMB2145(Cys4Ala)), YPNMB2145H30AFW-YPNMB2145H30ARP (for mutant pNMB2145(His30Ala)), YPNMB2145C34AFW-YPNMB2145C34ARP (for mutant pNMB2145(Cys34Ala)) and YPNMB2145C37AFW-YPNMB2145C37ARP (for mutant pNMB2145(Cys37Ala)). Mutations were confirmed by sequence analysis.

### RT-PCR

RNA was isolated from meningococci grown to late mid log phase (OD_600 _1.0 -1. 5) using Rneasy^® ^Midi Kit (Qiagen). RT-PCR was done using SuperScriptIII (Invitrogen). Primer pairs used to investigate whether NMB2140 through NMB2145 is transcribed as a polycistronic operon are depicted in Fig. 1. Primer pair CT-MSR-01/CT-MSR-02 was used to investigate transcription of NMB0044. One single batch of cDNA generated from RNA isolated from H44/76 wt, H44/76 + pNMB2144, ΔNMB2145 and ΔNMB2145 + pNMB2145, grown in the absence and presence of IPTG, was used for transcriptional analyses of the *rpoE *operon and NMB0044.To investigate the effect of hydrogen peroxide, diamide and singlet oxygen on RpoE activity, RNA was isolated from midlog phase grown cells with and without exposure to the stress stimuli and primer pairs CT-MSR-01/CT-MSR-02 and 2144-01/2144-02 were used to investigate transcription of NMB0044 and NMB2144 respectively. RT-PCR of RmpM (NMB0382) using primerset CT-class4-1/CT-class4-2, was used as loading control. Sequence analysis was carried out to confirm the identity of the generated RT-PCR products.

### Cell fractionation

Meningococci were grown in broth until OD_600 _= 0.6-0.8, harvested by centrifugation (20 min at 5000 × *g*) and resuspended in 50 mM Tris-HCl (pH 7.8). Meningococcal cells were disrupted by sonication (Branson B15 Sonifier, 50 W, 10 min, 50% duty cycle, 4°C), followed by centrifugation (3000 × *g*, 4 min, 4°C). The supernatant was centrifuged (100,000 × *g*, 60 min, 4°C). This way obtained supernatant was considered as the cytoplasmic fraction and pellets, containing crude membranes were resuspended in 2 mM TrisHCL (pH 6,8). Protein concentrations were determined by the method described by Lowry [82,83].

### SDS-PAGE and MALDI-TOF mass spectrometry

Proteins were resolved by SDS-PAGE [84]. Gels (11%) were stained with PageBlue (Fermentas), washed in MilliQ water and stored in 1% acetic acid at 4°C until bands of interest were excised for further analysis. MALDI-TOF mass spectrometry was carried out as described previously [64].

## Authors' contributions

CThPH participated in the design of the study, carried out experiments and analyses of the data and helped to draft the manuscript. DS carried out the MALDI-TOF mass spectrometry and helped to draft the manuscript. AvdE participated in the design of the study, carried out the analyses of the data and helped to draft the manuscript. YP participated in the design of the study, carried out the analyses of the data and drafted the manuscript. All authors read and approved the final manuscript.
